# Dietary intake of Brazilian black and white men and its relationship to the bone mineral density of the femoral neck

**DOI:** 10.1590/S1516-31802006000500006

**Published:** 2006-09-07

**Authors:** Patrícia Constante Jaime, Maria do Rosário Dias de Oliveira Latorre, Alex Antonio Florindo, Tomoe Tanaka, Cristiano Augusto de Freitas Zerbini

**Keywords:** Bone density, Diet surveys, Diet, Men, Race relations, Densidade mineral óssea, Inquéritos sobre dieta, Dieta, Homens, Relações raciais

## Abstract

**CONTEXT AND OBJECTIVE:**

Osteoporosis and fragility fractures are an important public health problem. Although bone loss occurs with age universally, the incidence of bone loss fractures varies greatly between racial groups. The aim of this study was to examine the relationship between calcium, protein and energy intake and the bone mineral density of the femoral neck in Brazilian black and white men.

**DESIGN AND SETTING:**

This was a cross-sectional study, carried out in a teaching hospital in São Paulo.

**METHODS:**

The participants were 277 volunteer men, aged 50 years or older. The bone mineral density of the femoral neck (FNBMD) was measured by dual energy x-ray absorptiometry. The relationship between FNBMD and calcium, protein and energy intake, as assessed by a three-day food record, was analyzed using multiple linear regression models and was adjusted for age, height, physical activity and education level. The analysis was stratified by race (white and black).

**RESULTS:**

FNBMD presented similar means in the two racial groups (p = 0.538). Protein and energy intake did not show a significant correlation with FNBMD, either in the white or in the black population. Calcium intake showed a strong and independent correlation with FNBMD in the black men (partial r = 0.42).

**CONCLUSION:**

Calcium intake was a determinant of FNBMD for black men, aged 50 years or older, but not for the white ones.

## RESUMO

**CONTEXTO E OBJETIVO:**

Osteoporose é um importante problema de saúde pública. Embora a perda de massa óssea ocorra universalmente com a idade, a incidência de fraturas por fragilidade óssea varia largamente entre grupos raciais. O objetivo foi examinar a relação entre o consumo de cálcio, proteína e energia e a densidade mineral óssea (DMO) do colo do fêmur em uma população de homens brasileiros brancos e negros.

**TIPO DE ESTUDO E LOCAL:**

Estudo transversal, realizado em um hospital escola em São Paulo.

**MÉTODOS:**

Foram recrutados 277 homens voluntários, com 50 anos ou mais. DMO do colo do fêmur foi medida com um densitômetro de dupla emissão de raios-X. Os consumos de cálcio, proteína e energia foram avaliados pelo método de registro de três dias de consumo de alimentos. Foi analisada a relação entre DMO do colo do fêmur e os consumo de cálcio, proteína e energia, utilizando modelos de regressão linear múltipla, estratificados por raça branca e negra e ajustados por idade, altura, atividade física e escolaridade.

**RESULTADOS:**

DMO do colo do fêmur apresentou média semelhante nos dois grupos raciais (p = 0,538). Os consumos de proteína e energia não foram correlacionados com a DMO do colo do fêmur, tanto para os indivíduos da raça branca como da negra. Já o consumo de cálcio teve correlação forte e independente com a DMO do colo do fêmur nos homens negros (r parcial = 0,42).

**CONCLUSÃO:**

Concluímos que o consumo de cálcio foi um determinante da DMO do colo do fêmur destes homens negros brasileiros com idade maior ou igual a 50 anos, mas não para os homens brancos estudados.

## INTRODUCTION

Osteoporosis is an important public health problem because of the significant morbidity and mortality associated with bone fractures and the high cost of treatment.^1^ Osteoporosis and fragility fractures in men constitute a considerable health care burden.^2^ The risk of fracture is directly associated with bone mineral density (BMD).^3^ Bone loss is expected with age,^4,5^ and it dramatically increases the incidence of hip and vertebral fractures in both men and women.^6^ The frequency of occurrence of bone fractures in South America is lower than in other regions of the world; however, it is possible that this problem is becoming intensified due to the increasing life expectancy of the population on this continent.^7^

Although bone loss occurs with age for everybody, the incidence of bone loss fractures varies greatly between racial groups.^8^ Higher bone mass has been observed in black individuals than in white individuals.^9,10^

Calcium intake is a significant determinant of BMD. In countries with high osteoporotic fracture incidence, low calcium intake among older men and women is associated with increased fracture risk.^11^ On the other hand, inadequate protein intake, when too high or too low, increases the risk of osteoporotic fractures.^6,11^ Low body weight is related to bone mass decline and to increased risk of osteoporotic fractures.^12^ It should be noted that the majority of studies on dietary intake, bone mass and osteoporosis have been carried out in developed countries, where the population's lifestyle and food consumption are very different from those of developing nations. Moreover, although it is known that there are important differences in bone mineral density between black and non-black individuals, the study of dietary intake differences in racial groups has received little attention.

## OBJECTIVE

The aim of the present study was to determine the relationship between calcium, protein and energy intake and the bone mineral density of the femoral neck (FNBMD) in Brazilian men aged 50 years and older, comparing black and white individuals.

## METHODS

### Population

This was a cross-sectional study involving volunteer men. From February to August 1997, we recruited 307 healthy male subjects aged 50 years or older, all resident in the city of São Paulo (southeastern Brazil). They were men whose wives had been referred by their primary care physicians for a bone densitometry scan, or who had responded to a newspaper advertisement.

We excluded from the study men using any kind of medication or having medical conditions that could affect bone metabolism: there was one individual with cancer of the colon, one with multiple myeloma, one with hyperthyroidism and one with rheumatoid arthritis. Twenty-two individuals were excluded due to the lack of bone densitometry data and 25 individuals were excluded because they did not answer the dietary questionnaire. We also excluded the mulattos (n = 29) in order to better separate the white and black races, thus avoiding confusions caused by the effects of racial miscegenation or mistakes in racial classification.

Thus, the final sample consisted of 227 men. None were taking energy, calcium or protein supplements.

The present study was approved by the Ethics Committees of Heliópolis Hospital and the School of Public Health of the University of São Paulo, Brazil. All the participants provided informed written consent.

### Bone mineral density assessment

FNBMD was measured by dual energy x-ray absorptiometry (DEXA), using the Lunar 3.6z software (Lunar, Madison, Wisconsin, United States). We reported BMD as grams per square centimeter. The coefficient of within-subject variation for DEXA measurements was 1.5% for the femoral neck. The time taken to perform the examination was five minutes and the radiation dose emitted was minimal (0.01 µΣ/h).

### Dietary assessment

Calcium, protein and energy intake were assessed using the food record method for three days. Food intake was recorded at all the daily meals, over three non-consecutive days, including one weekend day (Saturday or Sunday). Once the data was collected, the food intake registered was converted into energy and nutrients using the Virtual Nutri program.^13^

### Anthropometric assessment

Weight (kg) was measured using a standardized balance-beam scale and height (cm) using a stadiometer, and the body mass index (BMI) was calculated as the ratio between weight (kg) and the square of height (m^2^).

### Other variables

The participants answered a questionnaire about sociodemographic characteristics, including age (years), education level (years) and race (white, black, mulatto and Asian).

The information was self-reported and collected by a research assistant.

Concerning physical activity, three scores of physical activity were calculated (leisure, locomotion and occupation) using the questionnaire proposed by Baecke et al.,^14^ which had been adapted and validated for the adult Brazilian population.^15^ For more details regarding the construction of the physical activity scores, refer to Florindo et al.^16^

### Statistical analysis

The nutrient intake (calcium and protein) was adjusted for the total energy consumption using the residual nutrient method.^17^ In summary, this method allows analysis of the net effect of the nutrient without the influence of the energy intake. Energy intake was adjusted for height.

We compared the means of FNBMD, calcium, protein and energy intake, height, weight, body mass index, age, physical activity scores and education level according to white and black race using the Student t test. The Kolmogorov-Smirnov test was conducted to assess goodness of fit to normal distribution and only the calcium intake was transformed to log values. The relationship between nutrient (calcium and protein) and energy intakes and FNBMD was analyzed using Pearson's correlation coefficient and multiple linear regression models, stratified by white and black race. FNBMD was considered to be the dependent variable and calcium, protein and energy intakes were the independent variables.

The control variables were: age (in years), height (in centimeters), education level (years) and physical activity (scores). The models were estimated by the stepwise forward procedure. The control variables were kept in the multiple regression model if they were statistically significant (p < 0.05) or if they adjusted the regression coefficients of the variables that had already been kept in the model by at least 10%. The correlation coefficients (*r_i_*) and the regression coefficients (β*_i_*) of the dietary variables were corrected by between and within-subject variation, in accordance with Beaton et al.^18^

The effective p value for observations to be considered statistically significant was 0.05. Data were analyzed using the SPSS (Statistical Package for the Social Sciences) statistical software system.

## RESULTS

Most of the men (89%) were classified as white (n = 246). We observed that the black individuals (n = 31) were relatively younger and presented lower education levels (2.14 versus 4.41 years; p = 0.007), lower calcium intake (557 versus 720 mg; p = 0.020) and higher physical activity scores at work (2.95 versus 2.72 score units; p = 0.028) in comparison with the white individuals, as shown in Table 1. The other variables, including FNBMD (p = 0.538), presented similar means in the two racial groups.

**Table 1:** Mean and standard deviation of study variables, according to white and black race

Variable	White men (n = 246) Mean (SD)	Black men (n = 31) Mean (SD)	p
FNBMD (g/cm^2^)	0.91 (0.14)	0.93 (0.14)	0.538
Calcium intake* (mg)	720.07 (346.33)	557.73 (235.93)	0.027
Protein intake* (g)	86.06 (16.61)	89.82 (18.71)	0.488
Energy intake (kilocalories)	1980.36 (529.73)	1800.53 (462.88)	0.068
Height (cm)	165.33 (6.49)	166.69 (7.03)	0.268
Weight (kg)	73.04 (12.83)	74.81 (13.58)	0.472
BMI (kg/m^2^)	26.68 (4.10)	26.80 (3.82)	0.883
Age (years)	62.60 (8.14)	59.71 (5.63)	0.052
Leisure PA (score)	2.63 (0.66)	2.49 (0.62)	0.256
Occupational PA (score)	2.72 (0.52)	2.95 (0.60)	0.028
Locomotion PA (score)	1.64 (0.58)	1.67 (0.44)	0.777
Education level (years)	4.41 (4.06)	2.14 (2.71)	0.007

* nutrient intake adjusted for the energy intake by using regression analysis; FNBMD = bone mineral density of the femoral neck; BMI = body mass index; PA = physical activity, SD = standard deviation.

There was no correlation between calcium, protein and energy intake and FNBMD in the white men. For the black men, calcium (r = 0.567; p < 0.001) and protein (r = 0.359; p = 0.040) intake showed a significant positive correlation with FNBMD. Age showed a significant negative correlation for both white and black individuals (Table 2).

**Table 2:** Correlation coefficient (r) between bone mineral density of the femoral neck (g/cm^2^) and other study variables, according to white and black race

Variable	Bone mineral density of the femoral neck			
	White men (n = 246)		Black men (n = 31)	
	r	p	r	p
Calcium intake* (mg)	0.001†	0.984	0.567†	< *0.001*
Protein intake* (g)	0.055†	0.505	0.359†	*0.040*
Energy intake (kilocalories)	0.034‡	0.600	0.066‡	0.350
Height (cm)	0.232	< 0.001	0.308	0.092
Age (years)	-0.228	< 0.001	-0.394	*0.028*
Leisure PA (score)	0.241	< 0.001	0.252	0.171
Occupational PA (score)	0.192	< 0.001	0.048	0.799
Locomotion PA (score)	0.180	< 0.001	0.326	0.073
Education level (years)	0.158	0.013	0.322	0.078

* nutrient intake adjusted for the energy intake by using regression analysis;

† Correlation corrected for the between and within- subject variation in nutrient intake in the racial category;

‡ Correlation adjusted for the height (r partial) and corrected for the between and within-subject variation in energy intake in the racial category.

In the final regression model for the white individuals, we observed that the regression coefficients for calcium (β = 0.00579; p = 0.926), protein (β = 0.00058; p = 0.299) and energy (β = −0.00000; p = 0.953) intake were not significant. Thus, we found that the determiners of FNBMD in the white men were height (β = 0.00419; p = 0.004), age (β = −0.02429; p = 0.040) and leisure physical activity (β = 0.05076; p < 0.001), as shown in Table 3.

**Table 3:** Multiple linear regression model for bone mineral density of the femoral neck (g/cm^2^) in Brazilian white men (n = 246)

Independent variables	β	p
Calcium intake* (mg)	0.00579†	0.926
Protein intake* (g)	0.00058†	0.299
Energy intake (kcal)	- 0.00000†	0.953
Height (cm)	0.00419	*0.004*
Age (years)	- 0.02429	*0.040*
Leisure PA (score)	0.05076	< *0.001*
Educational level (years)	0.00129	0.586
Adjusted r^2^	0.121 (p < 0.001)	
Partial r (calcium)	0.015 (p = 0.850)	

* nutrient intake adjusted for the energy intake by using regression analysis;

† β corrected for the between and within-subject variation in nutrient intake in the white men; PA = physical activity.

For the black men (Table 4), we observed that the determiners of BMD were calcium intake and age. We found that, among the dietary intake variables, only calcium had an independent effect (partial r = 0.42; p = 0.002). Neither protein and energy consumption, nor height, physical activity scores and education level were determiners of FNBMD. Although the education level was not a determiner of BMD in the multiple model (p = 0.586), nor did it adjust the regression coefficient values of the dietary variables, we decided to keep this variable in the final multiple model in order to control for educational differences between the racial groups. We decided to keep height in order to adjust the energy effect, and also as a control variable in relation to bone mass. However, the effect of the adjustment for the height on the energy intake regression coefficient was small (before the adjustment: β = 0.00008; p = 0.120; and after the adjustment: β = 0.00007; p = 0.160; data not shown in the table).

**Table 4:** Multiple linear regression model for bone mineral density of the femoral neck (g/cm^2^) in Brazilian black men (n = 31)

Independent variables	β	p
Calcium intake* (mg)	0.41727†	0.031
Protein intake* (g)	0.00192†	0.261
Energy intake (kilocalories)	0.00005†	0.425
Height (cm)	0.00261	0.517
Age (years)	-0.00793	0.054
Education level (years)	0.00792	0.421
Adjusted r^2^	0.36 (p = 0.008)	
Partial r (calcium)	0.42 (p = 0.002)	

* nutrient intake adjusted for the energy intake by using regression analysis;

† β corrected for the between and within-subject variation in nutrient intake in the black men.

Thus, we found that, despite the small number of black individuals (n = 31), the calcium intake correlated with FNBMD only in black men, both in Pearson's correlation analysis and in the multiple regression models.

## DISCUSSION

This was the first study to analyze the relationship between nutrient intake and bone mineral density in men from different races living in Latin America. We noticed that FNBMD did not differ between the racial groups and the same was true for weight, height and BMI. In contrast, higher bone mass has been reported in black individuals in comparison with white individuals.^8-10^ Higher BMD in black individuals may be associated with higher obesity and muscular mass rates.^19^ This was not observed in the white and black men who participated in this study, since they presented similar mean weight and BMI.

White race is considered to be a risk factor for low BMD, and little is known about racial differences that affect calcium intake. We found that the white individuals consumed more calcium than the blacks. Wang et al. studied young Americans of both sexes and found a lower relationship between calcium and energy in blacks than in whites and Hispanics.^10^

We found out that calcium intake was a determinant of FNBMD only for the black men. Other studies conducted among adult men found a relationship between current calcium intake and bone mass.^20,21^ However, the effect of calcium intake on bone mass is still controversial. In contrast, calcium intake was not associated with whole body, hip and lumbar spine BMD in a study carried out among adult men in Canada.^22^

Both in the white and black men, protein and energy intake were not correlated with FNBMD in our study. Wang et al.^10^ also found no correlation between calorie, protein and calcium intake and FNBMD in Americans from different racial groups.^10^ Assessing another bone site, Whiting et al.^22^ found that, after energy adjustment, protein intake was a significant predictor of lumbar spine BMD in adult men.^22^ In analyzing the independent relationship between energy consumption and BMD, the action of body mass as a confounding variable needs to be considered. It is known that increased energy intake promotes increased weight and, in turn, this weight acts mechanically on the skeleton, thereby increasing BMD.^21^ Therefore, the effect of energy intake would be indirect and mediated by weight gain.

The analysis of associations between dietary variables and FNBMD may have been negatively affected in the present study by limitations on its cross-sectional design. Nevertheless, the results relating to the black race raise the hypothesis that there may be a mechanism for calcium action on bone structure that is different in black and white men. However, other studies are needed in order to clarify the protective effect of calcium intake on the BMD of black men, so that dietary intervention measures directed specifically towards the black population can be established.

## CONCLUSION

Calcium intake was a determinant of FNBMD for black men aged 50 years or older, but not for white men.
